# Elucidation of the Biotransformation Pathways of a Galnac_3_-conjugated Antisense Oligonucleotide in Rats and Monkeys

**DOI:** 10.1038/mtna.2016.31

**Published:** 2016-05-10

**Authors:** Colby S Shemesh, Rosie Z Yu, Hans J Gaus, Sarah Greenlee, Noah Post, Karsten Schmidt, Michael T Migawa, Punit P Seth, Thomas A Zanardi, Thazha P Prakash, Eric E Swayze, Scott P Henry, Yanfeng Wang

**Affiliations:** 1 Department of Pharmacokinetics and Clinical Pharmacology, IONIS Pharmaceuticals, Carlsbad, California, USA; 2 Structural Biology, IONIS Pharmaceuticals, Carlsbad, California, USA; 3 Medicinal Chemistry, IONIS Pharmaceuticals, Carlsbad, California, USA; 4 Toxicology, IONIS Pharmaceuticals, Carlsbad, California, USA

**Keywords:** antisense oligonucleotide, biotransformation, metabolism, *N*-acetyl galactosamine, targeted drug delivery

## Abstract

Triantennary *N*-acetyl galactosamine (GalNAc_3_) is a high-affinity ligand for hepatocyte-specific asialoglycoprotein receptors. Conjugation with GalNAc_3_ via a trishexylamino (THA)-C6 cluster significantly enhances antisense oligonucleotide (ASO) potency. Herein, the biotransformation, disposition, and elimination of the THA cluster of ION-681257, a GalNAc_3_-conjugated ASO currently in clinical development, are investigated in rats and monkey. Rats were administered a single subcutaneous dose of ^3^H-radiolabeled (^3^H placed in THA) or nonradiolabeled ION-681257. Mass balance included radiometric profiling and metabolite fractionation with characterization by mass spectrometry. GalNAc_3_-conjugated ASOs were extensively distributed into liver. The THA-C6 triantenerrary GalNAc_3_ conjugate at the 5′-end of the ASO was rapidly metabolized and excreted with 25.67 ± 1.635% and 71.66 ± 4.17% of radioactivity recovered in urine and feces within 48 hours postdose. Unchanged drug, short-mer ASOs, and linker metabolites were detected in urine. Collectively, 14 novel linker associated metabolites were discovered including oxidation at each branching arm, initially by monooxidation at the *β*-position followed by dioxidation at the *α*-arm, and lastly, tri and tetra oxidations on the two remaining *β*-arms. Metabolites in bile and feces were identical to urine except for oxidized linear and cyclic linker metabolites. Enzymatic reaction phenotyping confirmed involvement of *N*-acetyl-*β*-glucosaminidase, deoxyribonuclease II, alkaline phosphatase, and alcohol + aldehyde dehydrogenases on the complex metabolism pathway for THA supplementing *in vivo* findings. Lastly, excreta from monkeys treated with ION-681257 revealed the identical series as observed in rat. In summary, our findings provide an improved understanding of GalNAc_3_-conjugated-ASO metabolism pathways which facilitate similar development programs.

## Introduction

In the past decade, numerous advances have been underway to improve the pharmacology of antisense oligonucleotides (ASOs), which have included chemical modifications to the sugar bases, capping, and backbone, significantly improving ASO biostabilities. Mechanisms of action of ASOs are comprehensive and rely on the oligonucleotide chemistry, and other aspects, including the selected target mRNA binding site and may include RNase H degradation, 5′capping inhibition, and/or translation arrest.^1^ As of late, RNA therapeutics have garnered much attention and greater widespread clinical use is projected in the near future.^2^

Triantennary *N*-acetyl galactosamine (GalNAc) is a targeting ligand for ASOs which displays pronounced affinity and selectivity to the asialoglycoprotein receptor, a major cell surface receptor in hepatocytes. The design of GalNAc_3_ as a major targeting ligand is such that the oligonucleotide is functionalized with a tri-carbohydrate cluster in a tri-antennary format of established dimensions to allow recognition and enhanced binding affinity to facilitate hepatocyte uptake. GalNAc_3_-ASO conjugates (GalNAc_3_-ASOs) are administered subcutaneously with a rapid rate of uptake and eventual endocytotic clearance by the asialoglycoprotein receptor. A variety of GalNAc_3_-oligonucleotide conjugates with different spatial geometries have shown great promise effectively widening the therapeutic window for treatment of several genetic diseases.^3,4^ Targeted delivery using GalNAc_3_-conjugated ASOs has been shown to enhance hepatocyte potency greater than 10-fold in animals and likewise, numerous other investigations for RNA therapeutics exploit the GalNAc delivery platform to achieve enhanced receptor binding.^5,6,7,8^

In light of these observations, and based on internal data, previous animal studies revealed that GalNAc sugars and the trishexylamino (THA) cluster were rapidly cleaved within minutes to hours from GalNAc-conjugated ASOs following subcutaneous (SC) administrations, releasing unconjugated ASOs in liver to exhibit their pharmacological effects. It is well understood that unconjugated ASOs are slowly metabolized to chain-shortened oligonucleotide metabolites by nucleases and eliminated in urine.^9^ Hence, the distribution, metabolism, and fate of unconjugated ASOs are well established, yet the metabolic pathway of the THA cluster containing GalNAc_3_ as a targeting moiety has never been reported and is important to help understand the efficacy and safety of the conjugated ASO.

Metabolite proportionality and cross-species evaluations are essential for early drug development and assist preclinical safety studies that expedite clinical development.^10,11^ Such studies with radiolabeled drug for mass balance determinations remain a strong desire before new chemical entities expand into larger phase-3 clinical trials.^12^ As such, metabolic profiling offers early risk estimations and guides drug synthesis to improve stability, identify soft-spots of lead compounds, reduce *in vivo* degradation potential, and better ensure nonreactive intermediates, nonbioactivation, and reduce adverse drug reactions. Drug metabolism remains a critical issue for drug development and concerns regarding drug metabolism safety studies are warranted.^13,14^ Preclinical radiolabel mass balance studies remain invaluable and indispensable allowing a comprehensive and cohesive understanding of the *in vivo* behavior and action of drugs prior to exposure in large clinical studies.^15,16^ Clearly understanding the metabolic fate of 5′THA-GalNAc_3_-ASOs offers important insights into the extent of linker absorption, disposition, rate and routes of clearance mechanisms. Better assessments of the necessity for safety and/or relevant drug–drug interaction (DDI) studies are identified which lead development programs into a more complete path to overall product safety and successful clinical translation.

**Figure 1:**
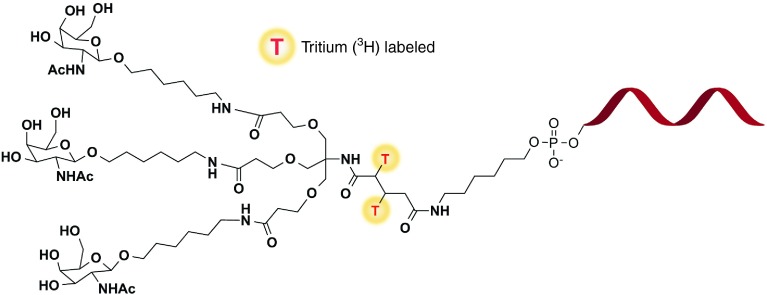
**Chemical structure of** ^**3**^**H-ION 681257 labeled on the THA linker**. T* denotes the tritium radiolabel position.

In the current work, for the first time, mechanistic investigations using both *in vivo* models and *in vitro* phenotyping to describe the role of disposition, metabolism and excretion and definite major elimination pathways of GalNac-conjugated ASOs are provided. Our report aims to provide a detailed account into (i) describing absorption, disposition, rate and route of elimination by mass balance, (ii) radiochromatographic profiling and characterization of metabolites by mass spectrometry, (iii) comprehensive identification of metabolizing enzymes, (iv) describing the overall biotransformation pathway, and lastly, (v) a cross species determination of common metabolic fate in rats and monkey. Herein, this study attempts to describe a comprehensive investigation into the biotransformation of the THA cluster on ION 681257, a 2′-O-(2-methoxyethyl) modified mixed PS/PO backbone ASO conjugated with a THA-C6-triantennary GalNAc_3_ at the 5′-end, targeting human lipoprotein (a) (Apo(a)) in rats and monkeys (**Figure 1**).

## Results

### *In vivo* mass balance and radiometric assessment of ^3^H-GalNAc-ASO (ION-681257)

**Figure 2:**
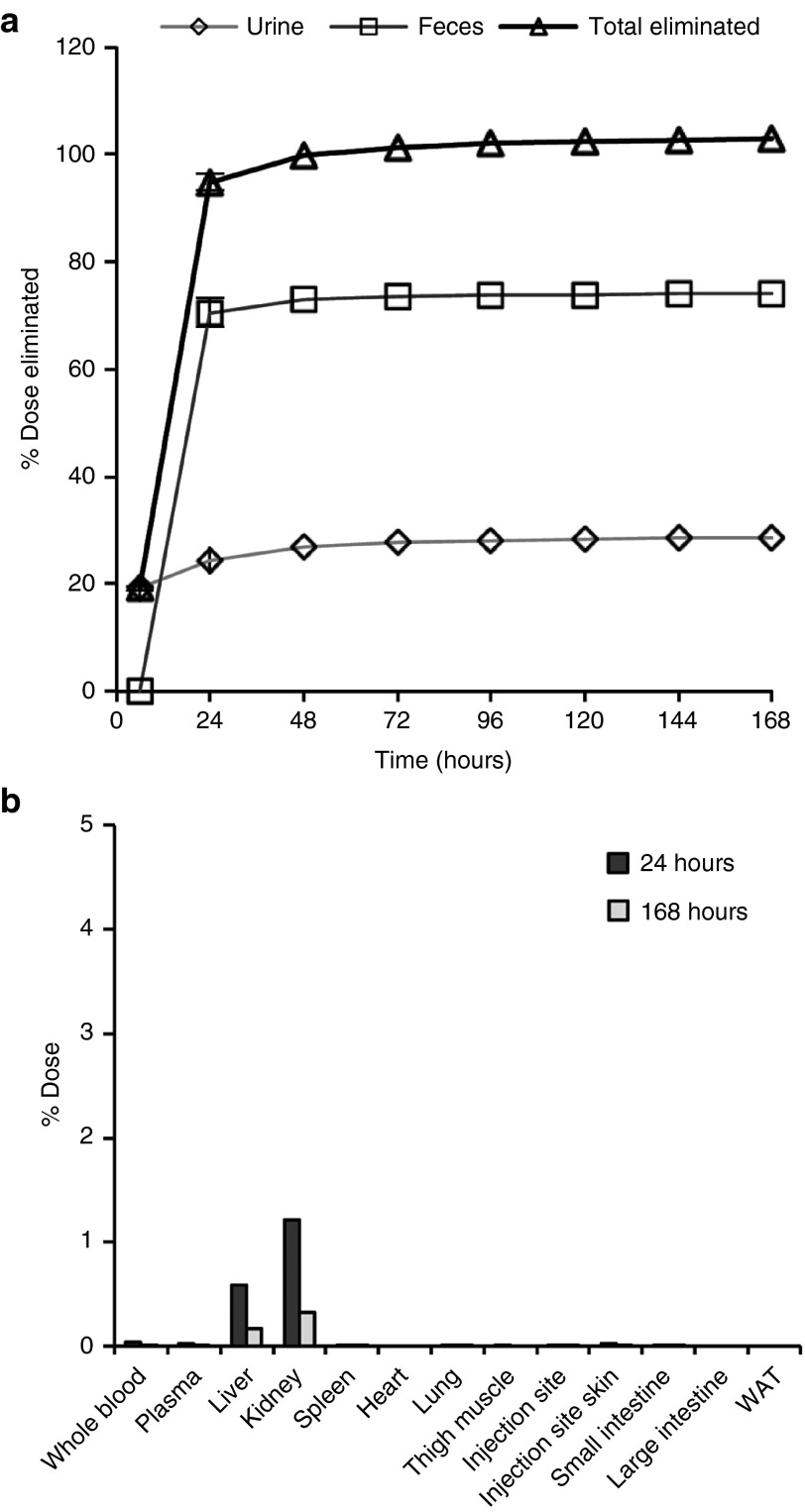
**Percent of dose eliminated in excreta and found in tissue**. (**a**) Mass percentages of cumulative radioactivity and total dose eliminated in Sprague Dawley rats following single s.c. administration of 4.3 mg/kg of ^3^H-ION 681257 labeled on the THA Linker at 114.4 µCi/kg (*n* = 2 rats, M). Values are listed as the percentage of normalized dose excreted in the urine, feces, and combined. (**b**) Percent of dose in major and minor tissues at 24 and 168 hours.

**Figure 3:**
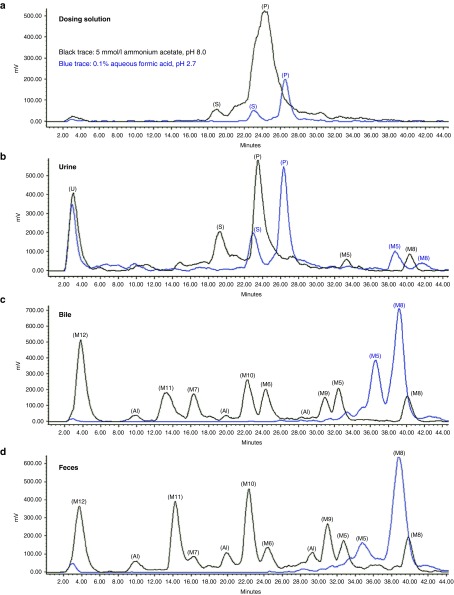
Representative HPLC radiochromatograms of (**a**) dosing solution, (**b**) rat urine, (**c**) rat bile, and (**d**) rat feces. Conditions of the mobile phase evaluated include (black trace) 5 mmol/l ammonium acetate pH 8.0 and (blue trace) 0.1% aqueous formic acid pH 2.7. All samples were diluted to 100,000 DPM per 20 µl injection. DPM, disintegrations per minute; M#, identified metabolite listed in **Table 1**; P, parent; S, short-mer; U, unidentified.

Mass balance (percentages) of cumulative radioactivity following a single SC dose of ^3^H-ION 681257 and tissue distribution are listed in **Figure 2**, (*n* = 2 rats). By total mass balance and compared with the percentage of normalized dose excreted in urine and feces it was found that 25.67 ± 1.635% and 71.66 ± 4.17% of the radioactive dose were accounted for by renal and biliary excretion, respectively. Radioactivites in both whole blood and plasma including both a 24- and 168-hour timepoint made up less than 0.1% of the cumulative radioactivity. Rapid disposition was evident with nearly complete excretion by 24 hours. As observed in **Figure 2b**, <3% of dose remained in major tissues (liver and kidney) at both 24 and 168 hours (*n* = 2 rats). Sample excreta including urine and feces were extracted and diluted to100,000 disintegrations per minute per 20 µl injection and assayed by radio-high-performance liquid chromatography (HPLC) detection using both basic and acidic mobile phase conditions. Metabolites observed contained ionizible functional groups evident at varying pH conditions. Chromatographic separation and near baseline resolution for most of the major metabolites were obtained using a shallow reversed phase gradient with most all species eluted prior to the introduction of (35%) acetonitrile in the gradient (**Figure 3**). The intact dosing solution profile was not altered by pH; however, mostly for all of the metabolites pH-dependent effects evident by a shift in retention time across the chromatogram were observed (**Figure 3a**–**d**).

The radiochromatographic profile for urine was distinguishable from bile and feces with a few similar shared peaks toward the end of the gradient, however, bile and feces were very near identical. Two significant peaks for bile and feces were obtained under acidic conditions (blue trace, representing 0.1% aqueous formic acid pH 2.7). However, using a basic buffer system (black trace, 5 mmol/l ammonium acetate pH 8.0), metabolites were substantially more polar and resolved well. Fraction collection of all of the major metabolite species was then performed for both the basic and acidic buffer system to evaluate the total distribution of radioactivity for each purified species. The pH-dependent separation determined by radiochromatography was also confirmed by liquid scintillation counting (LSC) of the fractionated metabolites. Bile duct cannulation was also examined to evaluate bile excreta from 0 to 2 hours, and 6 to 8 hours to determine the relative rate of metabolite formation.

### Non-radiometric evaluation and isolation of excreted metabolites for LC–MS

A separate experiment using male Sprague Dawley rats was investigated with rats divided into four dosing groups for single-dose SC administration of saline, 7.5, 30, or 100 mg/kg of nonradiolabeled ION 681257. Urine and feces were collected between 0 and 6 and 6–24 hours postdose. Following fractionation of all major species using the same chromatographic separation as described previously, each separate metabolite fraction was assayed by liquid chromatography mass spectrometry (LC–MS). As revealed in the mass spectra of urine extracts, the major species was determined to be intact ASO-conjugate, identical to that in the dosing solution. The full mass spectra charge envelope for the intact parent conjugated oligonucleotide was observed at various charge states ([M−H])^−^ with an averaged full mass corresponding to *m*/*z* 8,635. An abundance of nuclease cleaved species into short-mer oligonucleotides was also observed and corresponded to major ([M−H])^−^ mass to charge values of *m*/*z* 2,867, 3,187, and 3,532. The remaining material was accounted for by an early eluting species; although unidentified, we suspect a small and highly polar fragment of the THA linker.

Following fraction collection of bile and fecal samples of the high dose group, metabolites were assayed by LC–MS using a shortened gradient. High abundance masses were determined with similarities compared across each fraction of both bile and feces. Unique masses not present in control samples were tabulated. Major ions ([M+H])^+^ representing the major species (889.5, 937.5, 875.5, 923.5, 861.5, 909.5, and 903.5) were extracted and combined into a single chromatogram (see **Supplementary Figure S1**) with a clear pattern of major mass shifts of *m*/*z* ±14 and *m*/*z* ±48 present.

### Excreted metabolite profiling, characterization, and assignment

**Figure 4:**
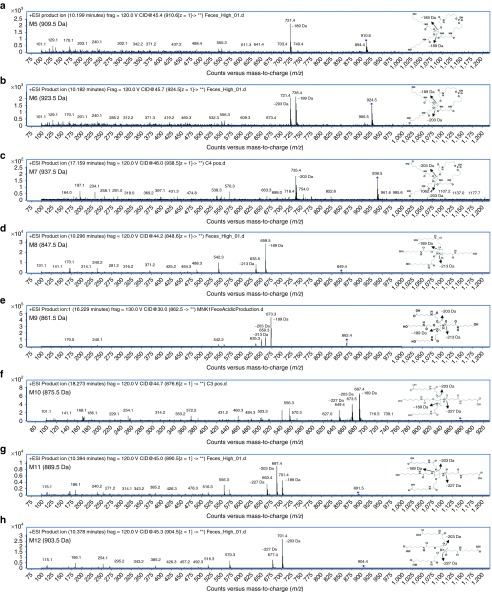
**Representative *in vivo* metabolism of the major GalNAc**_**3**_**-associated metabolites identified in rat feces**. MS/MS product ion spectra (**a**–**h**) represent metabolites (M5–M12) and are listed with *x*-axes representing *m*/*z* values, with the *y*-axes as relative abundance (counts).

The major metabolite series was evaluated using MS/MS fragmentation to obtain individual product ion spectra and fragmentation patterns (**Figure 4a**–**h**). In several cases, a distinctive similarity in the product ion spectra depicting common fragmentation ions was detected. A major nonoxidized cyclized linker (M5) relating to the THA cluster was observed to fragment to [M+H]^+^ in ESI (+) at *m*/*z* 721.4, corresponding to a loss of 189 Da while monooxidized cyclic linker (M6) is observed to have two large fragment ions with losses of both (189 and 203 Da) corresponding to both the loss of a single nonoxidized and monooxidized branching arm (positions A and either b_1_–b_3_). Major metabolite (M7), a dioxidized linker, was shown to have a major loss of 203 Da alone (**Figure 4a**–**c**) corresponding to fully oxidized branching arms as terminal oxidations to carboxylic acids. Product ion spectra of the proposed cyclic metabolites were seemingly much more resistant to ion bombardment even at higher collision energies. Product ion spectra patterns are comparable for the noncyclized metabolite series and focused on the cleavage of various branching arm substituents from the major cluster (**Figure 4d**–**h**). Noncyclic metabolite series from (M8) are feasible due to the presence or absence of distinctive fragmentation ions including losses of (189, 203, 213, and 227 Da) in the series. Of note, when each substituent arm is fully oxidized the loss of 189 Da is absent (**Figure 4h**). Product ion spectra reveal the initial oxidation on either of the b_1_–b_3_ branching arms, while the second oxidation occurs at position a_1_ while the third and fourth oxidations occur on either of the b_1_–b_3_ positions.

### *In vitro* GalNAc_3_ cleavage by *β*-*N*-acetylglucosaminidase

**Figure 5:**
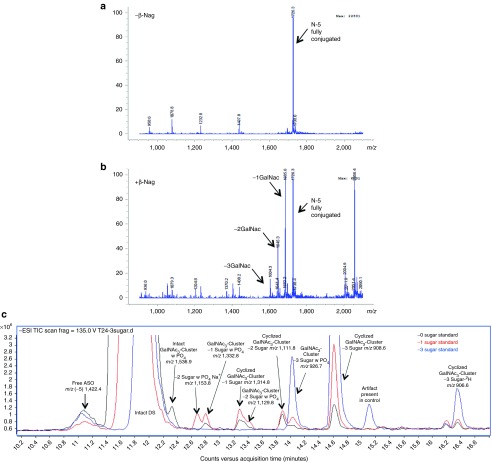
**Representative HPLC–MS chromatograms depicting *in-vitro* cleavage of GalNAc sugars from conjugated oligonucleotide (ION-681257) using *β*-*N*-acetylglucosaminidase and DNase II**. Reaction conditions include (**a**) 100 µmol/l ION-681257 without *β*-Nag was used as a negative control, and (**b**) 100 µmol/l with 122 U/ml of *β*-Nag. Reactions were incubated for 30 minutes at 37 °C. The mass spectra illustrate rapid cleavage of all sugars from the intact drug substance. (**c**) Neat standards corresponding to −0 sugar (black trace), −1 sugar (red trace), and −3 sugars (blue trace), respectively, were diluted in water to 0.32 mg/ml and incubated with 0.12 mg/ml of DNase II at 37 °C for 24 hours.

Enzymatic incubation of the intact parent GalNAc_3_-ASO conjugate with *β*-Nag was performed to confirm rapid sugar cleavage. Following a 30-minute incubation using 122 U/mL of *β*-Nag in the reaction, mass spectra reveal a rapid cleavage and loss of −1 (M1), −2 (M2), and −3 (M3) sugars from the cluster (**Figure 5a**,**b**). The intact drug substance in absence of *β*-Nag revealed a fully conjugated and intact GalNAc_3_–ASO complex with [M−H]^−^ in ESI (−) revealed a major ion (*m*/*z* 1,726.3) corresponding to the −5 charge state observed in the full mass spectra. However, upon addition of *β*-Nag in the rapid incubation, [M−H]^−^ masses corresponding to −1 sugar (M1) (−5 *m*/*z* 1,685.6), −2 sugars (M2) (−5 *m*/*z*, 1,645.3), and −3 sugars (M3) (−5 *m*/*z* 1,604.3) were observed, each as a distinctive loss of a sugar from the intact parent conjugate (P) (−5 *m*/*z* 1,726.3).

### *In vitro* DNase II digestion of GalNAc conjugated and unconjugated ASOs

Neat standards consisted of both intact-conjugated parent GalNAc_3_-ASO (−0 sugar), in addition to −1 and −3 sugar standards corresponding to the conjugated-ASO absent two or all GalNAc moieties, respectively. Neat standards were incubated in the presence of DNase II for 0, 4, 8, and 24 hours to evaluate cleavage of the oligonucleotide from the cluster. From the total ion chromatogram depicted in (**Figure 5c**) for the −0 sugar standard, following incubation with DNase, the intact conjugate is observed with the presence of numerous metabolites. The mass spectra reveal the cleavage of the oligonucleotide from the GalNAc_3_ cluster. High abundance full mass [M−H]^−^ in ESI (−) revealed a (−5) *m*/*z* ion, 1,422.4, corresponding to the free ASO. The cleaved GalNAc_3_ cluster was observed (*m*/*z* 1,536.9) in addition to numerous sugar deletions of the linker cluster including the GalNAc_3_ cluster with −1 sugar deletion (*m*/*z* 1,332.8), and −2 sugar deletions (*m*/*z* 1,129.8). Moreover two high abundance peaks also observed in excreta extracts corresponded to two distinctive metabolites observed as *m*/*z* 926.7 corresponding to the linear (M8) with PO_4_ (M4), and *m*/*z* 908.6 as the hydrolyzed and stable cyclic linker (M5). Following incubation with DNase II for the −1 and −3 sugar standards, the chromatographic profile was quite different. The major products observed included the intact parent (−4) *m*/*z*, 2,005.1, free ASO (−5) *m*/*z* 1,422.3, and both metabolites identified from the previous set (M4) *m*/*z* 926.7, and (M5) *m*/*z* 908.6, this time in much greater abundance. Following peak abundance assignment for the various species in (**Figure 5c**) it was found that the −0, −1, and −3 sugar standards formed esterified cyclic species 4.7-, 4.3-, and 6.0-fold greater than linear linker containing PO_4_ (M5 compared with M4, respectively).

### *In vitro* hydrolysis of GalNAc ASOs using alkaline phosphatase

**Figure 6:**
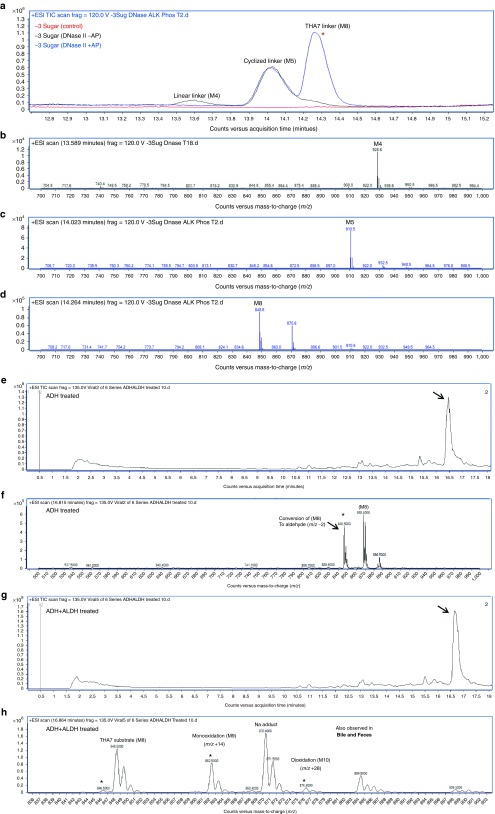
**Representative HPLC–MS chromatograms depicting *in-vitro* metabolism of GalNAc**_**3**_**-conjugated oligonucleotide (ION-681257) using DNase II treatment followed by alkaline phosphatase, alcohol, and aldehyde dehydrogenases**. (**a**) Combined samples including the −3 sugar standard absent enzyme as a control depicted in the total ion chromatogram. (**b**) Mass spectra of control samples following DNase II treatment, but absent alkaline phosphatase revealed the presence of linear metabolite (M4). (**c**, **d**) Mass spectra of the −3 sugar standard treated by DNase II for 18 hours and incubated with alkaline phosphatase for 2 hours depicting (M8) in high abundance in addition to residual cyclized linker (M5). ALH and ALDH treatment using (M8) standard alone (**e**–**h**). (**e**) Total ion chromatogram of (M8) treated with alcohol dehydrogenase, and (**f**) corresponding mass spectra. (**g**) Total ion chromatogram of (M8) treated with alcohol dehydrogenase followed by aldehyde dehydrogenase and corresponding mass spectra (**h**). Both mono and di-oxidations are observed.

Following incubation with DNase II using a neat −3 sugar standard, samples were protein precipitated and dried down. Substrates were then reconstituted in 1 mol/l diethanolamine buffer 0.40 mmol/l MgCl pH 9.8 and incubated with 10 U/ml of alkaline phosphatase for 2 hours at 37 °C. The total ion chromatogram of samples including time 0 controls is depicted in **Figure 6a** with major peaks of interest near 14.2 minutes. Mass spectra of the −3 sugar standard treated with DNase II alone is observed in (**Figure 6b**) depicting the major formation of both M4 and M5. DNase II treatment followed by alkaline phosphatase treatment results are observed in (**Figure 6c**,**d**) and observation of [M+H]^+^ in ESI (+) reveals both the formation of *m*/*z* 848.6 (M8) linker in high abundance and the presence of cyclized linker *m*/*z* 910.5 (M5), both of which were also observed in urine, bile, and feces. These high abundance masses were likewise not observed in the corresponding control (**Figure 6a**).

### *In vitro* generation of oxidized metabolites using alcohol and aldehyde dehydrogenases (ADH and ALDH)

Metabolite (M8) standard was investigated as a potential substrate of alcohol dehydrogenase for oxidation into an aldehyde, which is further oxidized by ALDH. The initial reaction included an incubation of metabolite standard (M8), 1 mmol nicotinamide adenine dinucleotide (NAD+), with 76.4 U of ADH incubated for 2 hours at 37 °C. From the total ion chromatogram in **Figure 6e**,**f**, a [M+H]^+^ in ESI (+) mass shift of *m*/*z* 2 was revealed, representing the oxidation of (M8) standard substrate (*m*/*z* 848.5) into the subsequent aldehyde (*m*/*z* 846.5). A separate reaction was run in parallel with the products from the initial ADH reaction reconstituted directly into 50 mmol/l 4-(2-hydroxyethyl)-1-piperazineethanesulfonic acid (HEPES) containing 1 mmol NAD+ and 1.4 U/ml of ALDH. Following a brief 2-hour incubation, a significant reduction of (*m*/*z* 846.5) and formation of both mono (*m*/*z* 862.5) and dioxidations (*m*/*z* 876.4) representing M9 and M10, respectively, were observed in the corresponding mass spectra (**Figure 6g**,**h**). It is important to note that the oxidations occur on any of the b_1–3_ positions of M8.

### Metabolite evaluation in monkey and accumulation study

A cross species comparison of monkey was evaluated following a 12 mg/kg SC administration of GalNAc_3_-conjugated ASO, ION 681257 and feces were collected from 12 to 24 hours. The chromatographic profile revealed an initial series of oxidations (M5–7) corresponding to 909.5, 923.5, and 937.5 Da, respectively (see **Supplementary Figure S2**). In addition the similar ion pattern was observed in ESI (+) revealing the identical *m*/*z* ± 48 mass shift at *m*/*z* ± 14 intervals corresponding to metabolites (M8–12), 847.5, 861.5, 875.5, 889.5, and 903.5 Da. No accumulation of any of the metabolite series was observed in plasma following the multiple dosing regimens.

## Discussion

**Figure 7:**
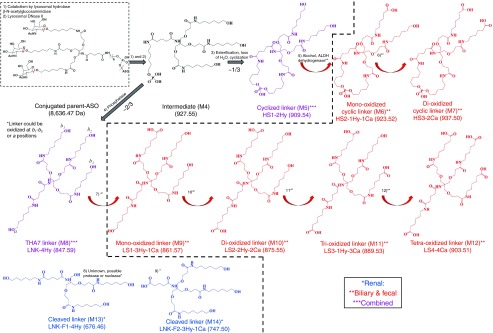
**Proposed major metabolism pathways of GalNAc_3_-conjugated antisense oligonucleotides**. Metabolites were detected in rat urine (blue, single asterisk), bile and feces (red, two asterisks), and/or combined (violet, three asterisks). The proposed biotransformation pathway is a result of characterization by LC–MS, including identity of precursor mass, product ions, retention times, and radiometric detection. An *in vitro* approach using enzymatic incubations was also used to confirm metabolite formation. From the scheme, it is observed that the full mass conjugated parent is rapidly metabolized into predominately oxidized species.

In this study, we have characterized the disposition and metabolism of ION-681257, a GalNAc_3_-conjugated-2′-MOE (methoxyethyl) ASO in rats, and monkey. To the best of our knowledge, to date this is the first report for GalNAc_3_ biotransformation (**Figure 7**). A rapid clearance of metabolites within the first 24 hours allowed effective *in vivo* and *in vitro* characterizations facilitating the identification of 14 novel GalNAc-ASO linker associated metabolites by radiometric and/or spectroscopic methods. Based on the fragmentation of metabolites, a common biotransformation pathway involving multiple oxidations was discovered. Apart from providing convincing evidence that helps us understand the elimination and disposition mechanisms and identify key metabolizing enzymes, and initial insight into the *in vivo* metabolic fate provides knowledge of potential pharmacological activity, cross species metabolite susceptibility, and overall metabolite safety.

Our initial assessment revealed the presence of intact parent ASO-linker-conjugate in urine with minor linker related metabolites, along with significant metabolites and no parent conjugated or free ASO in bile and feces. We next sought to collect bile and evaluate metabolite excretion in bile to identify and distinguish from those formed in the gastrointestinal tract and excreted in feces. For this approach, bile duct cannulated rats were used to reveal the rate of metabolite formation and order of appearance. Seemingly the rate of metabolite formation in bile of (M5–7) from 0 to 2 hours was maximal compared with (M9–11). Numerous less abundant peaks were observed in the bile and feces radiochromatograms, likely as nonfully metabolized aldehyde intermediates related to (M5 and M8). In urine, a large abundance of intact, nonmetabolized, parent GalNAc-conjugated-ASO corresponding to (47.1 ± 3.76%) of urine radioactivity), (*n* = 4) samples obtained from (*n* = 2) rats, was discovered in addition to the presence of short-mer oligonucleotides (15.0 ± 3.38%). As expected, short-mer formation from the ASO occurred rapidly as a result of 5′ and 3′-exo and endonuclease mediated metabolism seemingly more prevalent due to excess unbound free drug.^17,18,19,20^ Additionally, a pair of nonoxidized linker metabolites (M5 and M8), (3.65 ± 0.30%, and 5.67 ± 1.34% of urine radioactivity) and a single unidentified species (28.6 ± 2.35% of urine radioactivity) were discovered. Despite several attempts including varying column chemistries and chromatographic conditions, the single unidentified species in urine remained unaccounted for yet composed 7.35 ± 0.60 of total radioactivity by dose excreted. We might suspect the role of esterase activity on an amide bond of the conjugate forming a very polar and low molecular weight species, yet this is unconfirmed. We revealed the cyclized linker formation (M5) by an internal esterification and loss of water from linear intermediate (M4) from the intact parent, we also determine alkaline phosphatase responsible for generation of (M8). As a major accumulating and elimination organ, and in similar s.c. dosing studies in rats using^3^H-radiolabeled ASOs, the liver has been shown to contain the greatest proportion of dose administered.^21^ Thus, we suspected a large role of hepatic metabolism for THA linker and discovered major bile and feces metabolites including (M5) and (M8) (also observed in urine) and a novel and unique series of oxidized metabolites as (M6–M7) and (M9–M12). A pattern with both a *m*/*z* ± 48, and *m*/*z* ± 14 mass shift in the spectra represented both the light- and heavy-oxidized series. The major mass spectral differences for the oxidized metabolite series were accounted for by the number of oxidations on each branching arm of the cluster with the total possibility of major ions representing losses of 189, 203, 213, and 227 Da.

Following *in vivo* studies, next we sought to undertake an extensive effort to characterize and phenotype GalNAc metabolizing enzymes *in vitro* to reveal the contribution of a multitude of enzymes responsible for the *in vivo* biotransformations. An extensive evaluation of GalNAc sugar metabolism on the linker cluster was performed *in vitro* using *β*-*N*-acetylglucosaminidase and following *β*-Nag incubation, a rapid, specific, and extensive removal of sugar moieties from the intact conjugate was observed. Initially the fully conjugated GalNAc_3_–ASO complex remained intact, however, following incubation with *β*-Nag, loss of sugars was rapidly formed including the −1 (M1), −2 (M2) and −3 (M3) sugars. DNase II revealed cleavage of the intact linker cluster from the oligonucleotide. DNase II incubation of the intact and minus sugar standards also revealed the formation of a reactive linear intermediate (M4) which we propose may undergo internal esterification and loss of H_2_O forming a cyclized linker (M5), also observed in urine, bile, and feces. The cyclized linker formation for the −0, −1, and −3 sugar standards had ~4.7, 4.3, and 6.0 fold higher abundances for the formation of esterified species (M5) compared with the linear intermediate (M4), (*m*/*z* +18) over 24 hours, respectively. We revealed that DNase II cleaves the oligonucleotide selectively leaving the −PO_4_ moiety on the linker cluster liberating free ASO.

Reaction phenotyping using DNase II in an initial reaction followed by alkaline phosphatase revealed the formation of (M8) from linear linker (M4). Following DNase II incubation alone the presence of (M8) was negligible due to absence of alkaline phosphatase to hydrolyze (M4) into (M5) for (M8) formation. The profile revealed the prehydrolyzed linear linker (M4) as responsible for (M8) catalyzed by alkaline phosphatase. Following elimination of the GalNAc moiety from the linker as well as clevage of the linker cluster from the oligonucleotide, we demonstrated that alkaline phosphatase may generate major metabolite (M8) also seen in the urine, bile, and feces.

Our final phenotyping study confirmed that alcohol dehydrogenase metabolized (M8) into an aldehyde prior to oxidation by ALDH. Metabolite (M8) endured a loss of *m*/*z* 2 units due to the conversion of (M8) to an aldehyde using alcohol dehydrogenase. Using this newly formed substrate in subsequent reactions with ALDH formed both mono and dioxidized metabolites (M9 and M10). We suspect the *in vitro* formation of tri and tetra-oxidized species to be much less abundant and more difficult to observe. Although not confirmed *in vitro*, we presume that (M5) can be further oxidized into metabolites (M6 and M7) as observed *in vivo*. Based on our findings, the conversion to an aldehyde by ADH and further oxidation by ALDH may generate the metabolites (M9–M12) and (M6–7) observed in the bile and feces of rats and monkey feces.

Lastly, we were able to confirm that the major metabolites formed in rat are also present in monkey feces, suggesting a similar primary pathway of metabolism.^22^ This observation is also in line with similar animal and human model evaluations for unconjugated 2′-O-(2-methoxyethyl) modified ASOs which likewise demonstrate strong consistency of PK/PD relationships across species.^23,24^ Comparisons of metabolite exposures in both rat and monkey evaluating proportionality allow better confidence for safety coverage in preclinical species.

Ultimately multiple dosing studies provide essential information into accumulation properties and elucidate the necessity for drug–drug interaction studies. However, based on the short retention and rapid elimination of the 5′THA-GalNAc_3_-ASO-associated metabolites, with no detectable accumulation in plasma, we anticipate a much reduced potential for pharmacological activity and relative toxicity, with improved safety. Our drug metabolism research brings us strategically closer to complying with regulatory guidances regarding metabolite safety assessments set by the US Food and Drug Administration, in addition to the European Medicines Agency.^25,26^

Taken together, this study revealed that ION-681257, a GalNAc_3_-conjugated MOE ASO was well absorbed, rapidly distributed into liver tissues and exhibited extensive metabolism of the parent compound and linker conjugate. Based on the cumulative excretion of tritiated linker-related material in urine and feces, animals revealed at least 91.5% of dose was absorbed within the first 24 hours of dosing. An insignificant amount of radioactivity (<1%), and no major metabolites were recovered in circulation. Sugar deletions, oligonucleotide cleavage, and the formation of numerous oxidized species from the cluster were observed prior to biliary excretion (~71%) and renal excretion (~25%) following SC administration. Urine extracts contained the same metabolites except for those most extensively oxidized. Several GalNAc–ASO-conjugate metabolizing enzymes were identified *in vitro* including *N*-acetyl-*β*-glucosaminidase, DNase II, alkaline phosphatase, and alcohol and ALDH, each with seemingly similar metabolites formed from our *in vivo* findings. A total of four novel metabolites (M5, M8, M13, and M14) were identified in the urine, and eight in the bile and feces (M5–M7, M8–M12). The primary clearance mechanism of linker in rats was found to be attributed to phase-1 oxidations on the terminal position of each branching arm of the linker cluster. The current study is the first account of 14 different metabolites detected both *in vitro* and/or *in vivo* with a similar metabolic profile obtained for two animal species.

## Materials and methods

*Materials.* Potassium phosphate was purchased from Corning Life Sciences (Manassas, VA). HPLC-grade water, acetonitrile, methanol, formic acid, ammonium acetate, ammonium bicarbonate, dimethyl sulfoxide, ethylenediaminetetraacetic acid (EDTA), phenol:chloroform:isoamyl alcohol (25:24:1), triethylamine, hexafluoroisopropanol, triethylammonium bicarbonate, *N*-acetyl-*β*-glucosaminidase from jack beans, deoxyribonuclease II from porcine spleen (*Lot 029K7360V*), alkaline phosphatase (*Lot SLBL7932V*) from bovine intestinal mucosa, acid phosphatase (*Lot 053M4028V*) from potato, alcohol dehydrogenase (*Lot SLBH7396V*) from saccharomyces cerevisiae, and ALDH (*Lot BCBK5982V*) from bakers yeast were purchased from Sigma-Aldrich (St. Louis, MO). NAD+ (*Lot 01*) was purchased from Selleck Chemicals LLC (Houston, TX). Chlorox bleach was obtained from VWR Scientific (Seattle, WA). Luna 3 μm C18 (2), 2.0 × 150 mm, and 96-well Strata X polymeric reversed phase SPE cartridges (33 µm) were purchased from Phenomenex (Torrance, CA). Xbridge column (50 × 2.1 mm, 2.5 μm) was purchased from Waters (Milford, MA). Ultima Gold XR scintillation cocktail was purchased from PerkinElmer Life Science (Waltham, MA). Ultra free-MC 0.22 µm GV durapore centrifugal filter units were purchased from Merck Millipore (Tullagreen, Ireland). Gimble mount swivel holder, 12-inch tether, dual luer swivel, quick connect dual harness, extension line (swivel to vial), and injection caps were purchased from SAI Infusion Technologies (Libertyville, IL).

*Drug substance synthesis and radiolabeling.* GalNAc_3_-conjugated 20mer ION 681257 targeting human lipoprotein (a) (Apo (a)) containing a 2′-O-(2-methoxyethyl) modified mixed phosphorothioate, phosphodiester (PS/PO) backbone was internally radiolabeled with [^3^H] incorporated into the glutaric acid moiety of the stable THA-C6 cluster and conjugated at the 5′ end. [^3^H]-ION 681257 (m.w. 8,632.4 Da) was synthesized in-house at Ionis Pharmaceuticals (Carlsbad, CA) with a specific activity of 35.91 μCi/mg and a radiochemical purity of 90% determined by radio-HPLC. To minimize the potential for^3^H_2_O exchange, drug substance was formulated and mass balance studies were initiated within a week following synthesis. Chemical structure of the compound and tritium radiolabel position on the conjugate is provided in **Figure 1**. The comprehensive structure–activity relationship of the nonradiolabeled *N*-acetylgalactosamine-conjugated ASO has been described.^27^ Authentic standards of nonradiolabeled material including intermediate metabolite (M8), (–1, and –3 GalNAc_3_)-conjugated ION 681257 in addition to intact GalNAc_3_-conjugated 681257 were all synthesized in-house as materials used for chromatographic and mass spectrometric assessment during the study, structures provided in **Table 1**.

**In vivo* mass balance and radiometric assessment of 3H-GalNAc3-ASO (ION 681257).* All animals and procedures conducted in the study were in compliance and approved by the Ionis Pharmaceuticals Institutional Animal Care and Use Committee (protocol: P-0226-100028). Male Sprague Dawley rats aged between 8 and 9 weeks (250–300 g) were obtained from Charles River Laboratories (Hollister, CA). The rats were maintained on a 12-hour light dark cycle housed at 23 °C with 70% humidity with access to food and water ad libitum. Rats were weighed the day of dosing and administered a single SC dose of ^3^H-ION (681257) at 4.3 mg/kg at a specific activity of 35.9 µCi/mg and total radioactivity of 38.2 µCi per rat using a dosing volume of 1 ml/kg at the nape of the neck. Three male rats were used in study with two dosed equally as described including one as a saline control. Rats were housed separately into assigned metabolic cages and urine, feces, and cage washes were collected separately including a predose, 0–6, 6–24, and every 24 hours up to 1 week for radiometric measurements. Whole blood was collected at 24 hours and 1 week at the time of necropsy in addition to major and minor tissues. Rats were anesthetized using isoflurane followed by euthanization by cardiac puncture and cervical dislocation. Whole blood was collected into K2 EDTA tubes and immediately centrifuged at 2,000*g* for 10 minutes to collect plasma. Weights of urine, feces, cage rinses, plasma, and tissues were recorded and a 100 µL or 100 mg aliquot of urine, cage wash, plasma, or tissue homogenate was added directly into liquid scintillation vials with the addition of 5 ml Ultima Gold XR scintillation cocktail for LSC. Whole blood was processed by weighing 100–150 mg into sctinillation vials with 2 ml of soluene 350 and 2 ml of isopropyl alcohol with incubation at 55 °C for 5 hours and cooled overnight. Then 550 µl of 30% H_2_O_2_ was added and incubated at 55 °C for 30 minutes, after cooling 100 µl was added into 10-mL cocktail buffer and light adapted for 4 hours. Feces were diluted 4× (w/v) with 200 mg in filter sterilized purified water and homogenized into a uniform paste. Samples were then decolorized, solubilized with 2 ml of Clorox bleach and incubated at 55 °C for 5 hours. After solubilization, a 100-µl aliquot was added directly to liquid scintillation vials with the addition of 10-ml Ultima Gold XR scintillation cocktail and were maintained in the dark overnight prior to LSC. Sample aliquots were further evaluated by HPLC with radiometric detection for bioanalytical method development to profile metabolites.

**In vivo* non-radiometric evaluation, dosing with GalNAc3-ASO (ION 681257).* Male Sprague Dawley rats (*n* = 4) between 250 and 300 g were divided into four dosing groups with one saline dosing vehicle control and one for each dose group at a 7.5, 30, and 100 mg/kg dose, respectively. Rats were dosed using a single SC administration using a dosing volume of 1 ml/kg and immediately housed into individual metabolic cages as described previously with access to food and water ad libitum. Urine and feces from each rat were collected between 0–6 and 6–24 hours postdose. At the end of the study, rats were sacrificed as previously described.

*Bile duct cannulated rat study.* Male Sprague Dawley rats between 250 and 300 g with bile duct and duodenal catheterizations were obtained from Charles River Laboratories with dual portharnesses. Five dosing groups were used including a saline dosing vehicle control, a 7.5, 30, and 100 mg/kg dose of ION-681257, respectively, in addition to ^3^H-ION (681257) dosed at 20 mg/kg with 60 µCi of total administered radioactivity. Predose bile was collected from each rat and all compounds were administrated as a single SC dose to the nape of the neck. For each rat (*n* = 1), a tether was attached to the bile port with the duodenal port capped for bile flow through an extension line held by a tether. The line was then passed through a swivel so that the outlet collection line outlet maintained below each rat with sampling tubes maintained on ice. Bile was collected from each rat in 2-hour intervals between 0–2, 2–4, 4–6, and 6–8 hours, respectively, with a collection rate of ~2.0 ml per hour. A 2 µl aliquot of each sample from the^3^H-ION (681257) dosing group was added in combination with 5.0 ml of scintillation cocktail for LSC to determine total radioactivity and percent dose excreted. At the end of the study rats were euthanized. See **Supplementary Materials and Methods** for additional details.

*Sample preparation and isolation of metabolites for LC–MS/radiochromatography.* Initial method development was performed using a Waters 2690 Alliance HPLC Separations Module, Waters (Milford, MA) coupled with a v2 radiometric detector, LabLogic (Brandon, FL). During radiometric profiling, all extracts including (urine, bile, and feces) were first assayed by liquid scintillation individually to obtain respective total radioactive counts. Samples were then diluted accordingly to standardize and normalize the radioactivity of each sample to 100,000 disintegrations per minute per 20 µl injection, or 5,000 disintegrations per minute/µl. A 100-µl aliquot of urine collected between 0 and 6 hours from both 4.3 mg/kg ^3^H-ION (681257) dosed rats containing roughly 500,000 disintegrations per minute was added to separate Ultra free-MC 0.22 µm GV durapore centrifugal filter units and centrifuged for 4 minutes at 10,000*g* at room temperature.Bile obtained from^3^H-ION (681257) dosed rats at 20 mg/kg with 60 µCi of total administered radioactivity was also processed in the same manner. All filtrates were removed and dried down under ambient temperature using a Speed-Vap evaporator, Horizon Technology (Salem, NH). An aliquot of feces from rats dosed with 4.3 mg/kg ^3^H-ION (681257) was weighed to 120 mg and 1.5 ml of extraction buffer containing (methanol and H_2_O), 80:20 volume by volume (v/v) was added.^28^ Approximately 100 mg of ceramic beads was added to each tube and samples were pulverized for 2 minutes. Tubes were then centrifuged at 2,000*g* for 5 minutes. The supernatant was removed, with all samples added to centrifugal units, and recentrifuged at 10,000*g* for 4 minutes, followed by filtrate evaporation. All samples (urine, bile, and feces) were then reconstituted with 100 µL of mobile phase at a 95:5 v/v solution of (A1 or A2) with B1, respectively, which consisted of either (A1), (0.1% aqueous formic acid, pH 2.7), or (A2), (5 mmol/l ammonium acetate, pH 8.0), with (B) acetonitrile, for both, respectively. A Luna 3μm C18 (2) 2.0 × 150-mm column maintained at 25 °C using an injection volume of 20 µl at an initial flow rate of 0.3 ml/minute was used. For chromatographic separation of metabolites, a gradient elution was setup programming 5% B from T0–2, 8% B at T8, 11% B at T14, 14% B at T20, 17% B at T26, 20% B at T32, 23% B at T38, 26% B at T44, 29% B at T50, 95% B at T50.01 with a 4-minute hold at 95% and a 10-minute equilibration at initial conditions. Fraction collection every 2-minute intervals using the same gradient was performed to further isolate and purify metabolites with the entire eluent from each fraction added to 5 ml of Ultima Gold XR scintillation cocktail for LSC to evaluate radioactivity.

*Profiling, structure elucidation, and characterization of metabolites by LC–MS.* Structure elucidations were performed in several ways including radio-chromatography, liquid chromatography mass spectrometry (LC–MS), and tandem mass spectrometry (MS–MS). Structure elucidation of metabolites was initially evaluated by scanning for a range of biotransformation possibilities.^29^ Individual fraction collected extracts, each consisting of 2-minute collected intervals were evaluated by LC–MS using an Agilent 1200 series binary pump liquid chromatography system interfaced with an Agilent 6520 Quadrupole Time-of-Flight Mass Spectrometer (QTof-MS), (Agilent, Santa Clara, CA).

Fractionated samples of bile, feces, and urine collected from both basic and acidic gradient conditions were dried down and reconstituted in initial mobile phase conditions followed by filtration using a 0.22-μm pore size centrifugal filter unit and centrifuged for 4 minutes at 10,000*g* with all samples injected and evaluated separately. The initial mobile phase for negative polarity ionization consisted of 25 mmol/l ammonium bicarbonate solution at pH 7.0 and acetonitrile, 95:5 v/v, and the flow rate was held at 0.25 ml/minute. A linear gradient at initial conditions was held for 0–2 minutes, followed by 10% B from 2 to 5min, 35% B from 5 to 15min, and 95% B from 15 to 16 min with a 2-minute hold, followed by a 6-minute reequilibration at 5% B with a total run time of 24 minutes. The same gradient was used for positive polarity ionization except that mobile phase (A) consisted of 0.1% aqueous formic acid. Electrospray ionization was used in both negative and positive ion mode, and source conditions were set as follows: drying gas (N_2_) temperature at 350 °C, and flow rate of 11 l/minute, nebulizer set at 45 psi, and capillary voltage set to 4,000 V. Initial analysis was carried out in full scan mode using a mass to charge (*m*/*z*) range of 350–2,500 with a scan rate of 2.0 spectra per second using a fragmentor voltage of 120 V with reference lock mass enabled at 5 psi.

Additionally an ion-pairing method was also evaluated for both dosing solution and urine fractionated extracts and included H_2_O with 50-μm EDTA as the reconstitution buffer. For ion pairing, chromatographic separation was achieved using an Xbridge column (50 × 2.1 mm, 2.5 μm) at a flow rate of 0.25 ml/minute using a gradient of (A) 15 mm TEA, 400 mm HFIP buffer pH 7.8 with (B) methanol, held at 15% B initially and ramped to 50% B to 9 minute, followed a second ramp for 1 minute to 70% B and maintained for 1 minute before returning to initial conditions with a 5-minute postrun using the same source conditions as described. Oligonucleotide related peaks and metabolites were evaluated in the total ion chromatogram and averaged for respective *m*/*z* values for high abundance ions. Multiple charged ion envelope for oligonucleotide related metabolites was observed with a common range of charged species up to a (−12) charge state.

MassHunter workstation software version B.06.00 of build 6.0.633.10, (Agilent, Santa Clara, CA) with molecular feature extraction was used to identify unique mass traces of metabolites common in each species compared with each fractionated matrix control sample. Potential compounds were background subtracted and initially filtered to restrict the number and ratio of elements and isotopic patterns in addition to peak abundance.^30,31^ Targeted tandem mass spectrometry (MS/MS) was then applied to sort five maximal high abundance precursor ions of each fractionated extract per cycle using a mass range of *m*/*z* 500–1500 at a scan rate of 1.5 spectra per second to obtain product ion spectra. An automated ramping collision energy table was used with *m*/*z* 400 with Z1 at 35, and Z2 at 30V, respectively, *m*/*z* 1,000 with Z1 at 60, and 55 V respectively, and *m*/*z* 2,000 with Z1 at 70, and Z2 65 V, respectively.

Radiochromatographic profiles were directly compared with the mass spectra of fractionated extracts of bile, feces, and urine, and of the controls, respectively. Metabolite masses in each fraction were compared with corresponding theoretical masses of biotransformed reference compounds. For most cases, a correlation of fragment masses of metabolites with proposed theoretical structures was compared with the MS/MS fragmentation patterns of available reference material (for example, M8). Proposed structures and MS/MS fragmentation patterns were compared with key fragments of the (M8) reference standard. To differentiate between both PO_4_ containing and non-PO_4_ containing metabolites a mass shift difference of *m*/*z* ± 48 was investigated in the spectra, presumably by internal esterification, loss of water, and cyclization.

*Nomenclature and assignment of metabolites.* Initially metabolite (M8) was characterized as an intermediate metabolite of the GalNAc_3_ cluster and used to assign other metabolites. Metabolites clearly identified by radiometric detection, chromatographic retention time, and mass spectral features were named in order of suspected formation (M1–M14). Metabolite (M8) contains four branching arms (A, b_1_, b_2_, and b_3_) with structural uniformity for the b_1–3_ series. Nonmetabolized parent compound is designated as (P) and pronounced but uncharacterized peaks are labeled as (X). Proposed structures and naming of all metabolites is described in **Table 1**.

**In vitro* GalNAc3 cleavage by *β*-*N*-acetylglucosamindase.* GalNAc_3_-conjugated ION 681257 was evaluated at 100 µmol/l and combined with a 100× dilution of *β*-*N*-acetylglucosamindase (*β*-Nag) at (122 U/ml) and incubated at for 30 minutes at 37 °C on a shaker set to 100 rpm. Reactions were terminated by freezing at −80 °C and samples were basified with NH_4_OH and extracted with phenol/chloroform/isoamyl alcohol (25:24:1) and centrifuged at 13000*g* for 4 minutes to separate aqueous and organic layers. Aqueous supernatants were removed and an additional back-extraction was performed. Following the liquid liquid extraction, solid phase extraction was performed. Solid phase extraction plates were equilibrated with 2 ml of acetonitrile followed by 2 ml of 1 mol/l triethylammonium bicarbonate. Samples were loaded onto the cartridges followed by a rinse with 2 ml of 0.1 mol/l TEAB, 2 ml of H_2_O, 2ml of 2% NH_4_OH (in 5% acetonitrile, 0.1 mol/l TEAB), and 2ml of 10% acetonitrile in 0.1 mol/l TEAB. Samples were eluted with 1.5 ml of 70% acetonitrile in 0.1 mol/l TEAB and run through a protein precipitation plate prior to LC–MS analysis.

*DNase II digestion of GalNAc conjugated and −3GalNAc-conjugated ASO.* GalNAc_3_-conjugated ION 681257 and −3GalNAc-conjugated ASO were diluted in water to 0.2 mg/ml, deoxyribonuclease II (DNase II) containing 757 U/ml was prepared at 0.4 mg/ml in a reaction buffer containing 20 mmol/l sodium acetate, pH 4.6, with 5 mmol/l MgCl_2_ as a cofactor. The digest was initiated by addition of 100 µl of DNase II to 100 µl of substrate. Samples were incubated for 0, 4, 8, and 24 hours at 37 °C. Reactions were terminated by the addition of 0.4 ml of ice-cold methanol with brief vortexing and centrifuged at 10,000*g* for 3 minutes followed by evaporation using a speedvac under ambient temperature. Samples were reconstituted in 100 µl of mobile phase and transferred to an autosampler vial for LC–MS under negative polarity electrospray ionization using a scan range of *m*/*z* 800–2,200.

**In vitro* hydrolysis study using alkaline phosphatase.* Lyophilized −3 sugar standard was reconstituted to form a 0.2 mg/ml solution in 20-mmol/l sodium acetate, pH 4.6, with 5 mmol/l MgCl_2_. To initiate the reaction, 75 µL of DNase II (0.4 mg/ml dissolved in the same buffer system) was added with 75 µl of the standards briefly mixed and incubated at 37 °C for 18 hours. Following digestion, 0.3 ml of ice-cold methanol was added, vortexed, and centrifuged at 10,000*g* for 3 minutes, the supernatant was removed and dried down. Stock alkaline phosphatase was diluted to 10 U/ml using 1 mol/l diethanolamine containing 0.50 mmol/l MgCl, pH 9.8, and 200 µl was added to the previous substrate directly. Samples were vortexed and incubated at 37 °C for 2 hours followed by the addition of 0.4 ml of methanol. Samples were mixed and centrifuged at 10,000*g* for 3 minutes. The supernatant was dried down and reconstituted in 100 µl of mobile phase (95:5, 0.1% aqueous formic acid, and acetonitrile, volume to volume, respectively). Positive polarity electrospray ionization using a −mass scan range of *m*/*z* 700–1000 was used.

**In vitro* generation of oxidized metabolites: enzymatic studies using ADH and ALDH.* As a potential substrate for alcohol dehydrogenase (ADH), M8 corresponding to the (THA linker) was prepared in 50 mmol/l HEPES pH 8.0 at 5.23 mmol. ADH (382 U/ml) 1.0 mg/ml and NAD+ at 1.0 mg/ml were both dissolved in the same buffer. The final reaction was set up using a total volume of 0.2 ml and included 123.5 µl of 50 mmol HEPES, 10 µl of 5.23 mmol M8 substrate, 26.5 µl of 1 mmol NAD+, and initiated with the addition of 40 µl ADH enzyme (76.4 U). Samples were incubated for 2 hours at 37 °C. Reactions were terminated with the addition of 2× volume of ice cold methanol, and vortexing. Samples were centrifuged for 3 minutes at 10,000*g* and the supernatant was dried down and reconstituted in mobile phase for LC–MS. Positive polarity electrospray ionization using a mass-scan range of *m*/*z* 500–1,000 was used Additional samples were processed in the same method but reconstituted in 50 mmol/l HEPES pH 8.0 and not mobile phase to be used as the substrate for ALDH. Potential metabolites M9–12 were carried over from the initial ADH incubation and used as the substrate with the addition of 26.5 µl of 1.0 mg/ml NAD+ and 173.5 µl of ALDH (1.4 U/ml). Samples were incubated for 2 hours at 37 °C, stopped, and assayed as described for ADH.

**SUPPLEMENTARY MATERIAL** **Figure S1.** Metabolites observed in rat bile (*n* = 2) as individually fraction collected and combined into a single chromatogram. **Figure S2.** Representative GalNAc_3_-associated metabolites confirmed in monkey feces by liquid chromatography mass spectrometry using positive polarity electrospray ionization following administration of 12 mg/kg ION 681257. **Table S1.** Mass balance with excretion route and percentage of total dose excreted. **Supplementary Materials and Methods**

## Figures and Tables

**Table 1:**
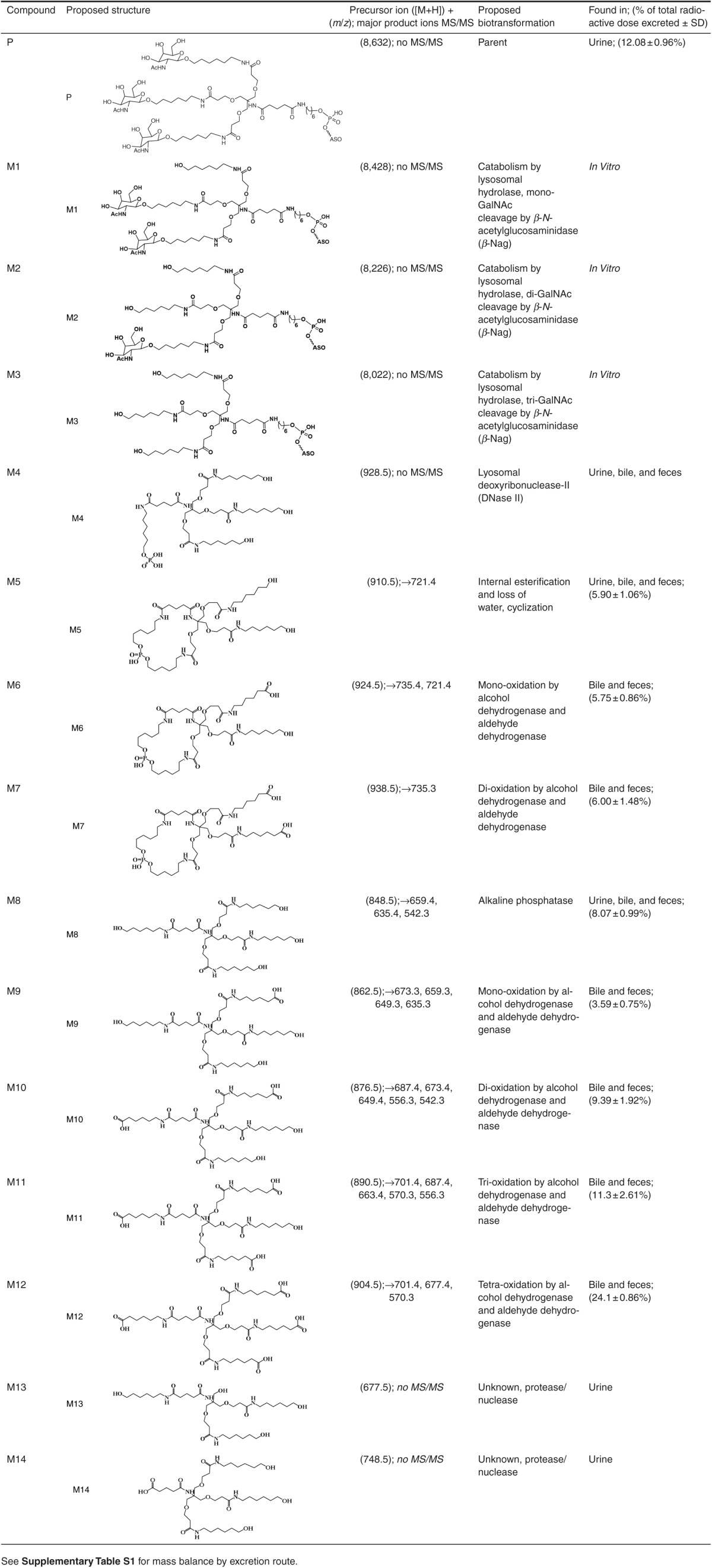
A summary of liquid chromatography tandem mass spectrometry (LC–MS/MS) data and proposed structures of GalNAc_3_-ASO metabolites observed in rat urine, bile, and feces and *in vitro* enzymatic reactions
