# Supplementary material for: Elucidation of the Biotransformation Pathways of a Galnac3-conjugated Antisense Oligonucleotide in Rats and Monkeys
Source: Mol Ther Nucleic Acids. 2016 May 10;5(5):e319–. doi: 10.1038/mtna.2016.31 (PMC5014515; doi:10.1038/mtna.2016.31)
Supplement: Supplementary Materials and Methods [file mtna201631x1.doc]

**Supplementary Table S1**

**Supplementary Table S1.** Mass Balance with excretion route and percentage of total dose excreted

| **Assayed Species** | **% Radioactivity Normalized in Urine** ± (SD) | **% Radioactivity Normalized in Feces**± (SD) | **% of Total Radioactive Dose Excreted** ± (SD) |
| --- | --- | --- | --- |
| **Unidentified** | 28.6 ± (2.35) | . | 7.35 ± (0.60) |
| **Shortmer** | 15.0 ± (3.38) | . | 3.85 ± (0.87) |
| **Parent** | 47.1 ± (3.76) | . | 12.08 ± (0.96) |
| **M5** | 3.65 ± (0.30) | 6.93 ± (1.37) | 5.90 ± (1.06) |
| **M6** | . | 8.03 ± (1.20) | 5.75 ± (0.86) |
| **M7** | . | 8.37 ± (2.07) | 6.00 ± (1.48) |
| **M8** | 5.67 ± (1.34) | 9.25 ± (0.89) | 8.07 ± (0.99) |
| **M9** | . | 5.00 ± (1.05) | 3.59 ± (0.75) |
| **M10** | . | 13.1 ± (2.68) | 9.39 ± (1.92) |
| **M11** | . | 15.7 ± (3.64) | 11.3 ± (2.61) |
| **M12** | . | 33.6 ± (1.19) | 24.1 ± (0.86) |

Values are listed as the mean ± standard deviation from (n=4) samples (n=2 rats) respectively. Metabolites M5 and M8 were combined for urine and feces to obtain total percent of dose excreted. Extracts in urine and feces were normalized to 25.7 ± 1.64%, and 71.7 ± 4.17% accordingly to mass balance respectively. Metabolites not listed were not readily distinguished by radiometric profiling, but were identified in cold non-radiolabeled studies.

**
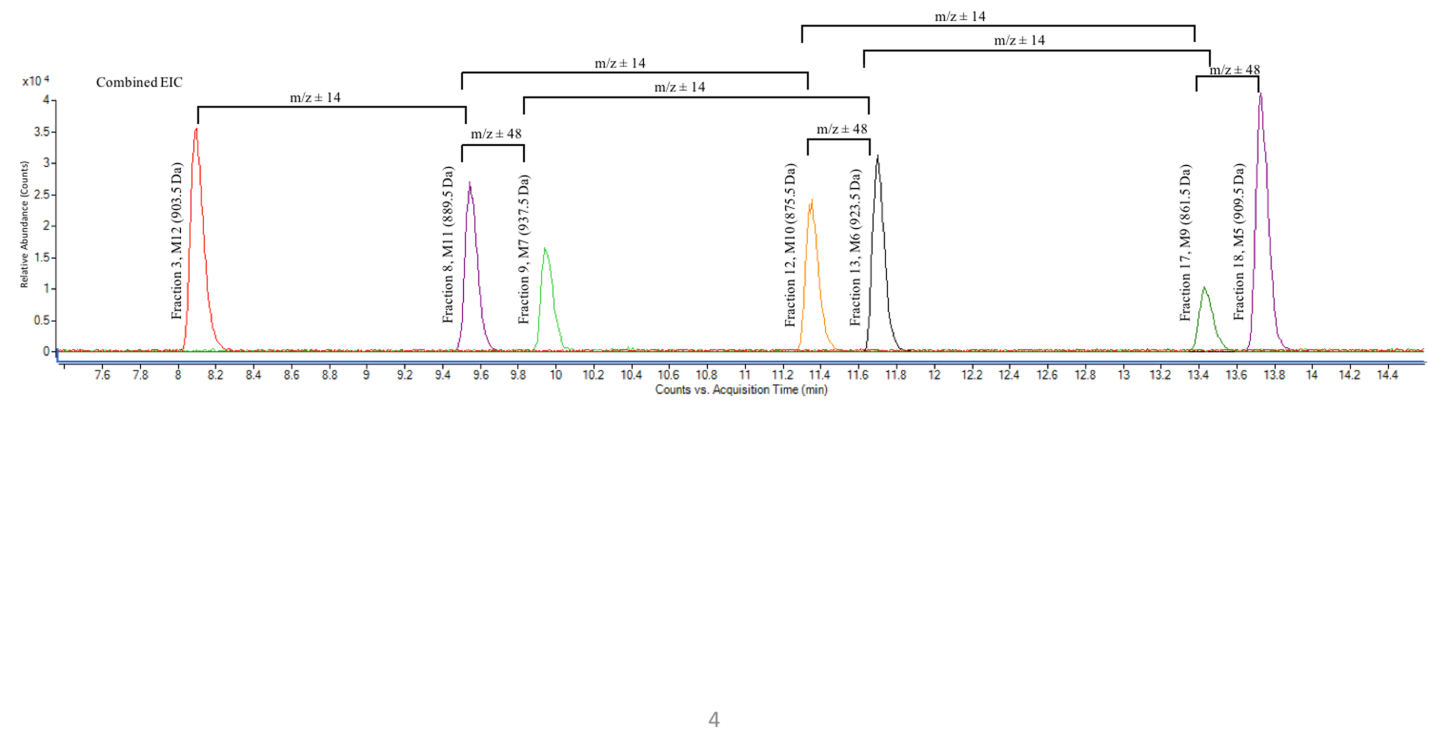
**

**Supplementary Figure S1.** Metabolites observed in rat bile (n=2) as individually fraction collected and combined into a single chromatogram. Numerous oxidized metabolites with mass shifts of *m/z* ±14, as well as phosphate cleavage differences *m/z* ± 48 observed.

**
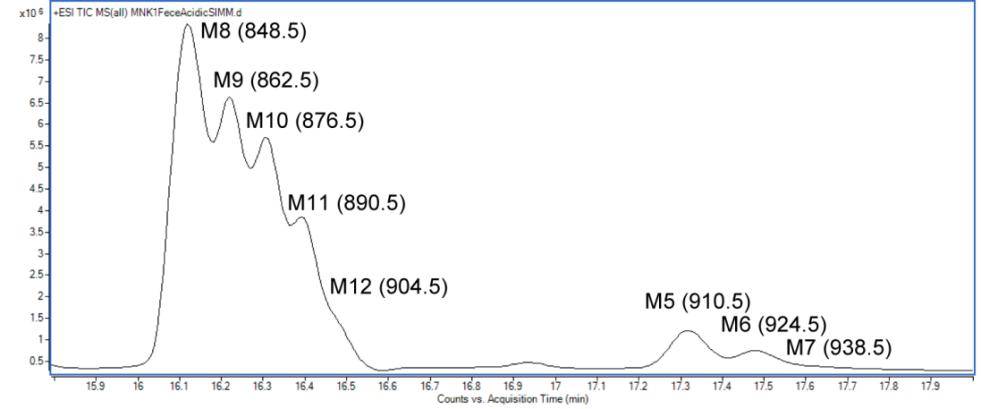
**

**Supplementary Figure S2.** Representative GalNAc_3_ associated metabolites confirmed in monkey feces by liquid chromatography mass spectrometry using positive polarity electrospray ionization following administration of 12 mg/kg ION 681257. Lack of baseline resolution for the metabolites was observed due to shortened gradient time for ease of analysis in addition to acidic buffer which minimizes the chromatographic separation. Initial series of oxidations are observed (M5-7) corresponding to 909.5, 923.5, and 937.5 Da, including the triplet series of signature ions ([M+H]) ^+^ containing a *m/z* ±48 mass shift and *m/z* ±14 sequences (M8-12) as 847.5, 861.5, 875.5, 889.5, and 903.5 Da.

**SUPPLEMENTARY MATERIALS AND METHODS:**

**Individually Fractionated Metabolites in Rat**

Two male Sprague Dawley rats (n=2) were administered a single SC dose of (100 mg/kg) ION 681257. Fractions were collected every 2 minutes, and individual purified extracts were injected onto a QTof-MS using positive mode electrospray ionization with a fullscan mass range of *m/z* 350-3000. Fractions were injected using a gradient elution with A) 0.1% aqueous formic acid and B) acetonitrile. For MS characterizations a shortened gradient was used from 0-5.0 min at (2% B), 5.0-15.0 min (35% B), 16.0-18.0 min (95% B), with a 6 minute post run at initial conditions. Ions ([M+H]) ^+^ representative of the major metabolites (890.5, 938.5, 876.5, 924.5, 862.5, 910.5, and 904.5) were extracted and combined into one chromatogram.

**Metabolite Evaluation in Monkey and Accumulation Study**

A male rhesus monkey (n=1) was administered a single SC dose of (12 mg/kg) of GalNAc_3_ ION-681257. Urine and feces was collected both pre-dose and between 0-24hours after dosing. Approximately 150 mg of feces was removed and extracted for LC-MS analysis as described previously with select ion monitoring of (M5-7) and (M8-12). Key metabolites associated to the GalNAc_3_ cluster oxidations were explored. Accumulation in plasma was evaluated following a q2d (every other day) loading regimen at 12mg/kg/wk administered subcutaneously for the first week on Days 1, 3, 5, and 7 for a total of 4 doses, followed by a q1w (once weekly) thereafter maintenance regimen on Days 14, 21, and 28.
